# Proteomics of severe SARS-COV-2 infection and paraquat poisoning in human lung tissue samples: comparison of microbial infected and toxic pulmonary fibrosis

**DOI:** 10.3389/fcimb.2024.1446305

**Published:** 2024-09-05

**Authors:** Jiang Min, Hou Jiaqi, Lin Lihua, Chai Qianqian, Wang Shujuan, Liu Xiang, Liu Liang, Ren Liang, Zhou Yiwu, Liu Qian

**Affiliations:** Department of Forensic Medicine, Tongji Medical College, Huazhong University of Science and Technology, Wuhan, China

**Keywords:** SARS-CoV-2, paraquat, PF, proteomics, autopsy samples

## Abstract

**Introduction:**

Pulmonary fibrosis (PF) encompasses a spectrum of lung conditions characterized by the abnormal accumulation of scar tissue in the lungs, leading to impaired respiratory function. Various conditions can result in severe PF, among which viral infections have emerged as significant triggers. In addition to viral infections, exposure to toxic substances such as paraquat represents another significant risk factor for PF. Therefore, this study aimed to explore the dissimilarities and similarities between PF triggered by viral infections and chemical toxicants, using the mechanism of PF in IPF as a reference.

**Methods:**

Data-independent acquisition proteomics technology was employed to identify COVID-19 and paraquat-induced PF from the autopsy of lung tissue samples obtained from individuals who died due to PF. Bioinformatics was employed for differential protein analysis, and selected indicators were validated on pathological sections.

**Results:**

Our results showed that the differential proteins associated with the two causes of PF were enriched in similar lung fibrosis-related signaling pathways, such as the Wnt signaling pathway. However, differences were observed in proteins such as CACYBP, we verified the consistency of the results with proteomics using the IHC approach

**Conclusion:**

This study illuminates distinct protein-level differences by investigating pulmonary fibrosis pathways in severe COVID-19 and paraquat poisoning. Although both conditions activate lung-protective and repair pathways, COVID-19 shows limited phosphorylation-independent ubiquitination of β-catenin compared to paraquat toxicity. These findings shed light on potential therapeutic targets for PF induced via diverse factors.

## Graphical Abstract

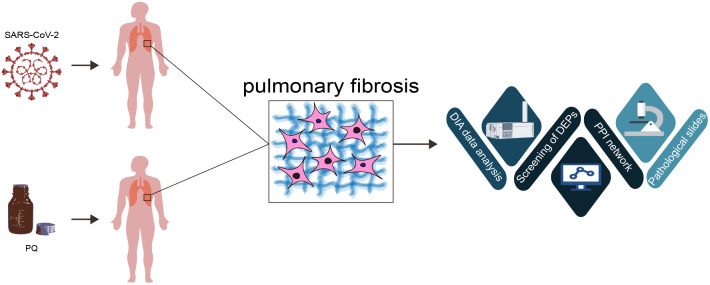

## 1 Introduction

Pulmonary fibrosis (PF) can occur owing to several factors that cause lung injury, including toxicity, autoimmune conditions, drug reactions, infections, and traumatic injuries (Thannickal et al., 2004). It begins with lung injury triggered by diverse factors, followed by the repair process of the damaged lung tissue. If hindered, the restoration of lung epithelial cells prompts macrophages to secrete profibrotic factors, stimulating the proliferation of fibroblasts and their transition into myofibroblasts. This results in the accumulation of extracellular matrix (ECM), culminating in the onset of PF (Vannella and Wynn, 2017; Hinz et al., 2007).

Viral infections, including CMV, Influenza, Avian influenza, SARS-CoV, and MERS-CoV, can induce PF. These infections present immediate risks and also persist as long-term risk factors contributing to PF following the resolution of the infection (Huang and Tang, 2021). Since October 26, 2023, the coronavirus disease 2019 (COVID-19), caused by severe acute respiratory syndrome coronavirus-2 (SARS-CoV-2), remains a significant global health concern, with approximately 775 million reported cases worldwide and 7.04 million deaths (WHO Coronavirus (COVID-19) Dashboard). In patients with severe cases of COVID-19, PF arises from the incomplete healing of lung damage caused by SARS-CoV-2 infection. This condition manifests as a form of pathological PF (George et al., 2020). Autopsy results from national and international sources consistently show a predominant lung pathology in patients who die following SARS-CoV-2 infection, with diffuse alveolar damage (DAD). During the proliferative phase of DAD, fibroblasts infiltrate the alveolar septa, developing interstitial and peribronchiolar fibrosis (Nalbandian et al., 2021; Liu et al., 2020a; Borczuk et al., 2020). The pulmonary fibrosis probably occurred due to SARS-CoV-2 infection of the host and damage to vascular endothelial and alveolar epithelial cells. Therefore, the body could not be completely repaired, resulting in the secretion of a large number of pro-fibrotic cytokines, proliferation of myofibroblasts and fibroblast, and significant increase in fiber collagen (type I, type III, type V, and type VI) and other ECM macromolecules (Rosenbloom et al., 2017).

Paraquat (PQ)—a potent herbicide known for its broad-spectrum activity and effectiveness—has extensive application in Indian agriculture. The Chinese government has implemented recent restrictions on its usage, and PQ poisoning remains a significant global public health concern that requires urgent attention (Jin, 2020; Kumar et al., 2021). A recent case report from Nepal highlighted the complex nature of acute PQ poisoning, which was compounded by the onset of PF (Yadav et al., 2023). Most fatalities resulting from PQ poisoning occur due to accidental or intentional ingestion of this toxic substance (Dinis-Oliveira et al., 2008). PQ ingestion can damage multiple organs or systemic functions, with the lungs being the primary target organ of PQ toxicity. This can result in early acute lung injury, progressing to PF. No effective antidotes are currently available for this condition (Dinis-Oliveira et al., 2008; Gawarammana and Buckley, 2011). Fei Gao et al. identified that *in vitro*, Wnt/β-catenin signaling pathway was activated and EMT was induced in A549 cells treated with PQ (Gao et al., 2020). Previous studies on PQ have relied on experimental animal models. However, studies investigating lung tissue from human participants are limited.

Many signaling pathways linked to human PF have been identified. However, this study primarily focused on idiopathic pulmonary fibrosis (IPF). Inhibiting the activation of the Wnt/β-catenin signaling pathway in mouse lungs via ICG-001 has shown effectiveness in PF (Henderson et al., 2010). Additionally, *in vitro* experiments involving alveolar epithelial type II (ATII) primary cells have confirmed the expression and functionality of Wnt/β-catenin pathway in adult lung epithelium (Konigshoff et al., 2008). The Annual Review of Pathology systematically describes the pathological mechanisms of IPF, highlighting the significant roles of Wnt and Notch signaling pathways (Moss et al., 2022). They presume that TGF-β acts as a master regulator of fibrosis (Moss et al., 2022).

This study utilized human lung tissue samples from autopsies at the Hubei Tongji Forensic Science Center. The sample comprised six patients who died of COVID-19, three of PQ ingestion, and three control patients who died of non-pulmonary diseases. To explore the distinctions between the two causes of PF, DIA proteomic analysis was conducted on the FFPE samples. This study aimed to identify proteins with differential expression associated with PF. Subsequently, clustering, GO, KEGG, and PPI analyses were conducted to elucidate the signaling pathways and proteins associated with PF. Additionally, HE staining was conducted on pathological sections, and immunohistochemistry was performed on selected markers to identify potential therapeutic targets for clinical treatment. Therefore, this study aims to investigate the differences between PF induced by viral infections and chemical toxicants, using the mechanism of PF in IPF as a reference to explore other potential mechanisms of pulmonary fibrosis; it provides a reference direction for the study of more specific drugs for the treatment of pulmonary fibrosis.

## 2 Materials and methods

### 2.1 Sample preparation

FFPE lung tissue samples from 12 autopsy sources were utilized to obtain lung histopathological sections. First, we selected patients with COVID-19 who died between February and March 2020, the period of the COVID-19 pandemic during which patients were primarily infected with the original SARS-COV-2 strain. For studying pulmonary fibrosis as a pathological manifestation, the effect of the original strain was noticeably more effective than that of omicron or other mutant strains. We also excluded other patient comorbidities that could affect the scientific soundness. In contrast, the deaths of patients with PQ occurred between 2018–2019, before the COVID-19 pandemic. This is a good way to avoid PQ data influencing COVID-19 data. Furthermore, the pathology of toxic pulmonary fibrosis caused by PQ poisoning was similar to COVID-19. Therefore, we selected PQ poisoning to compare with COVID-19. Finally, the control patients did not have any lung-related disease or injury, and the cause of death was mostly sudden cardiac death, and we considered these sample groups to be scientifically sound as a control group (**Supplementary Table 1**).

Proteomic samples underwent extraction and proteolysis processes. Lung autopsy samples in this study were provided by the Ethics Committee at Wuhan Jinyintan Hospital Ethics Committee (permission number: KY-2020-15.01) and Tongji Medical School, Huazhong University of Science and Technology (permission number: [2021] IEC-A001). Ethical approval for collecting samples from healthy donors were provided by the Ethics Committee at Tongji Medical School, Huazhong University of Science and Technology (permission number: 2022-S099). Written informed consent was obtained from the patient’s family before the autopsy.

### 2.2 Experimental design and statistical rationale

#### 2.2.1 DIA data analysis and quality control

Protein extraction and proteolysis were followed by high pH RP separation. DDA and DIA analyses were performed using nano-LC-MS/MS, which involved DDA library construction and DIA sample analysis. We used Fragepipe software to perform peak search and subsequently used the iRT peptide to correct the retention time.

#### 2.2.2 Database selection

Currently, databases in use can be categorized into three main groups: 1) The reference protein database, which is the most informative and resourceful protein database. 2) Protein databases based on Genome Annotations encompass several databases derived from the NCBI and Ensemble gene annotation databases. 3) Databases from other sources comprised newly generated gene sequences derived from genome or transcriptome sequencing for *de novo* assembly. Moreover, these databases may contain sequences featuring novel characteristics such as alternative splicing, mutation sites, and fusion genes.

#### 2.2.3 MSstats differential analysis

MSstats—an R package obtained from the Bioconductor repository (Choi et al., 2014)—facilitates statistical evaluation of significant differences in proteins or peptides across various samples. It finds extensive application in targeted proteomics, MRM, label-free quantitation, and SWATH quantitative experiments. The core algorithm utilized in this process was a linear mixed-effects model. Data preprocessing was conducted based on a predefined comparison group, followed by significance testing using the model. Subsequently, differential protein screening was performed, with a fold change of>2 and P-value <0.05 as criteria for significant differences. Simultaneously, enrichment analysis was carried out on differentially expressed proteins.

#### 2.2.4 Protein-protein interaction networks

We utilized STRING (functional protein association networks [string-db.org]) to analyze the interactions among PF-associated proteins. The obtained results were imported into Cytoscape _ v3.10.1 for visualization purposes.

### 2.3 Pathological slides

#### 2.3.1 Immunohistochemistry

Tissue slices underwent a series of steps for preparation and staining. Initially, the slices were sequentially placed in eco-friendly dewaxing solutions I and II for 20-min each, followed by soaking in anhydrous ethanol I and II, 75% alcohol, and water-ethanol I for 5 min each, before being washed with tap water. Subsequently, antigen retrieval was conducted by subjecting the tissue to EDTA (pH 9.0) in a microwave oven at medium heat for 8 min, followed by cessation of fire for 8 min, and a reduction to medium-low heat for 7 min. Endogenous peroxidase was blocked using 3% H_2_O_2_ for 30 min and sealed with 3% BSA for 2 h, 24°C. The primary antibody (anti-CACYBP: Boster, PB0965, 1:500) was incubated overnight at 4°C, washed, and subsequently incubated with the secondary antibody (horseradish peroxidase [HRP]-conjugated goat anti-rabbit IgG: ABclonal, AS014, 1:200) at 24°C for 50 min. DAB was added dropwise for 5 min to develop color and rinsed with tap water. Hematoxylin staining was performed for 3 min, followed by washing and differentiation, which returned to blue, and finally dehydrated to seal the film.

#### 2.3.2 Hematoxylin-eosin staining

The slices were sequentially placed in eco-friendly dewaxing solutions I and II for 20 min, anhydrous ethanol I and II for 5 min, followed by soaking in 75% alcohol, water ethanol I, and anhydrous ethanol II for 5 min, and a final-75% alcohol soak before washing it with tap water. The nucleus was stained with hematoxylin solution for 5-20 min. Subsequently, it was rinsed in running tap water. The slices were treated with Differentiation solution for 3 min, washed with tap water twice for 2 min each. They were then re-dyed with Eosin Y Aqueous Solution for 10 s to 2 min. Following this, they were dehydrated in alcohol (75%, 85%, 95%, 100% alcohol (I)), each for 2-3 s, and rinsed in 100% alcohol (II) for 1 min. Finally, they were made transparent using xylene and sealed with resinene.

#### 2.3.3 Sirius red stain

The slices were sequentially immersed in eco-friendly dewaxing solution I for 20 min, followed by eco-friendly dewaxing solution II for 20 min, anhydrous ethanol I for 5 min, anhydrous ethanol II for 5 min, and 75% alcohol for 5 min. They were subsequently washed with tap water. Next, the slides were stained with Sirius red staining solution for 8 min, followed by rapid dehydration using two or three cylinders of anhydrous ethanol. Subsequently, they were immersed in clean xylene for 5 min for clearing. Finally, the slides were dehydrated and sealed.

#### 2.3.4 Data analysis

Pathology microscopic slices were scanned using a Panoramic MIDI (3D HISTECH) scanner and visual fields were acquired. Twenty randomly selected fields of view per section were used for statistical analysis. Image J (Fiji Is Just) was used to calculate the mean optical density values and the results were imported into GraphPad Prism 9.5.0 for Dunnett-test.

## 3 Results

### 3.1 DEPs, GO, and KEGG

**Figure 1:**
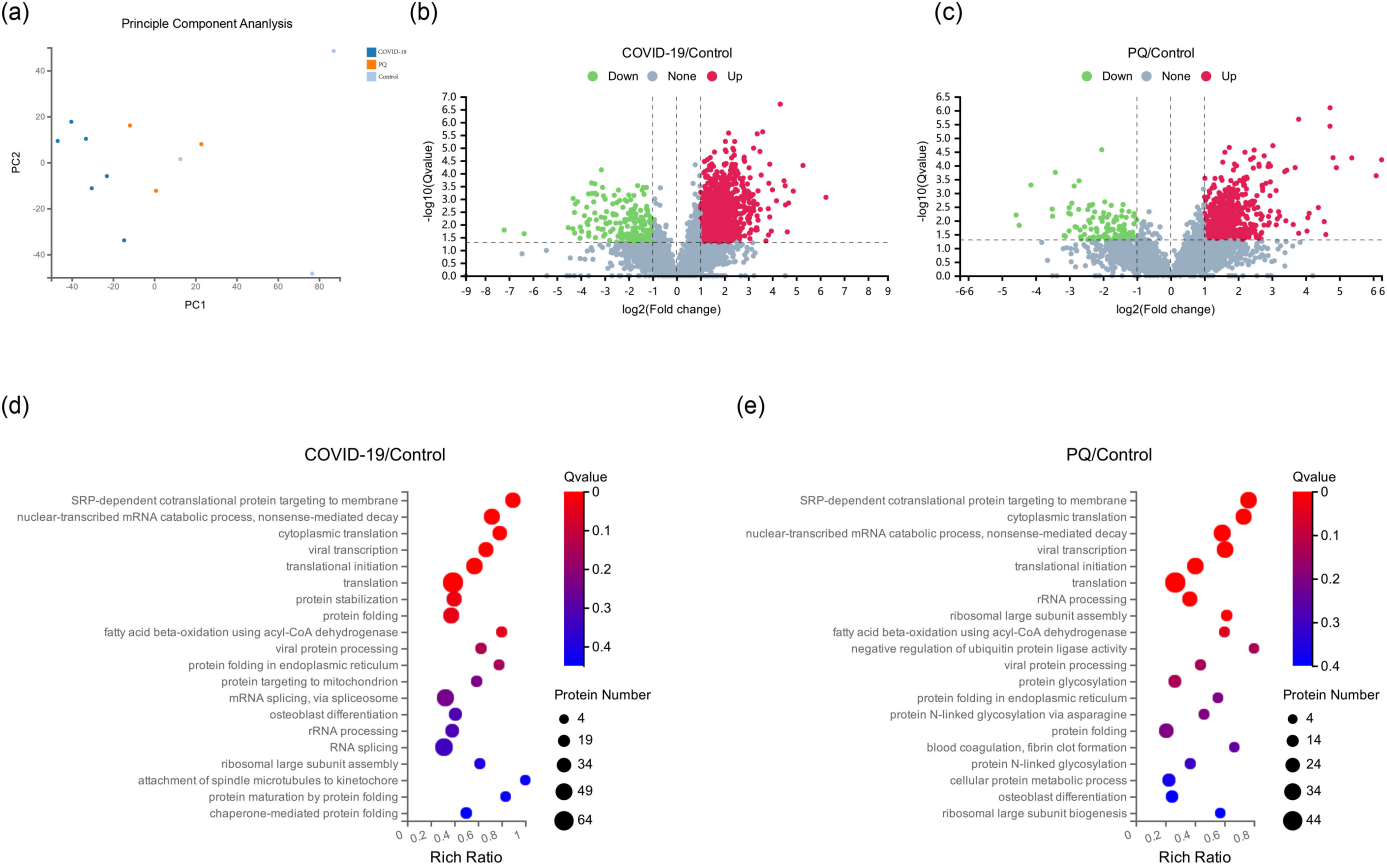
**(A)** Principal component analysis (PCA) of 12 pulmonary fibrosis samples. **(B, C)** Depicts volcano maps highlighting proteins detected in the two comparison groups. **(D, E)** GO enrichment analysis of two comparison groups in biological process.

Twelve human lung tissue FFPE samples obtained from autopsy cases at the Hubei Tongji Forensic Identification Centre were utilized in this study. Overall, 61,122 peptides and 6,251 proteins were detected in these samples using DIA proteomics (**Figures 1A**). Fold change ≥ 2 and Q value <0.05 were employed as screening criteria for significantly differentially expressed proteins (DEPs). Based on this criterion, 1,332 DEPs were identified between the COVID-19 and control groups, comprising 1,142 upregulated and 190 downregulated proteins. The number of DEPs between the PQ and control groups was 657, with 543 upregulated and 114 downregulated proteins. To compare the direct variability between the COVID-19 and PQ groups, rather than with the control group, we established separate COVID-19 vs. PQ comparisons. This analysis revealed 501 DEPs, consisting of 371 upregulated and 130 downregulated proteins (**Figures 1B, C**). Common biological processes included SRP-dependent cotranslational proteins targeting the membrane (GO:0006614), nuclear-transcribed mRNA catabolic processes, nonsense-mediated decay (GO:0000184), cytoplasmic translation (GO:0002181), viral transcription (GO:0019083), translational initiation (GO:0006413), and translation (GO:0006412). However, DEPs in the COVID-19 group were particularly enriched for protein stabilization (GO:0050821) and protein folding (GO:0006457). In the PQ group, the emphasis was more on rRNA processing (GO:0006364), ribosomal large subunit assembly (GO:0000027) and fatty acid beta-oxidation using acyl-CoA dehydrogenase (GO:0033539). In the PQ group, we focused on the negative regulation of ubiquitin-protein ligase activity (GO:1904667) enriched in four DEPs: RPS15, RPS20, RPL5, and BAG2. In the COVID-19 group, a significant biological process was mRNA splicing, which was enriched with 45 DEPs. Catenin Beta Like 1 (CTNNBL1) exhibited specific upregulation in the COVID-19 comparison group (log2FC=1.41, Q value=0.03). However, the difference was insignificant in the PQ comparison group (log2FC=0.85, Q value=0.20). It was enriched in mRNA splicing through the spliceosome (GO:0000398) cellular process (**Figures 1D, E**).

**Figure 2:**
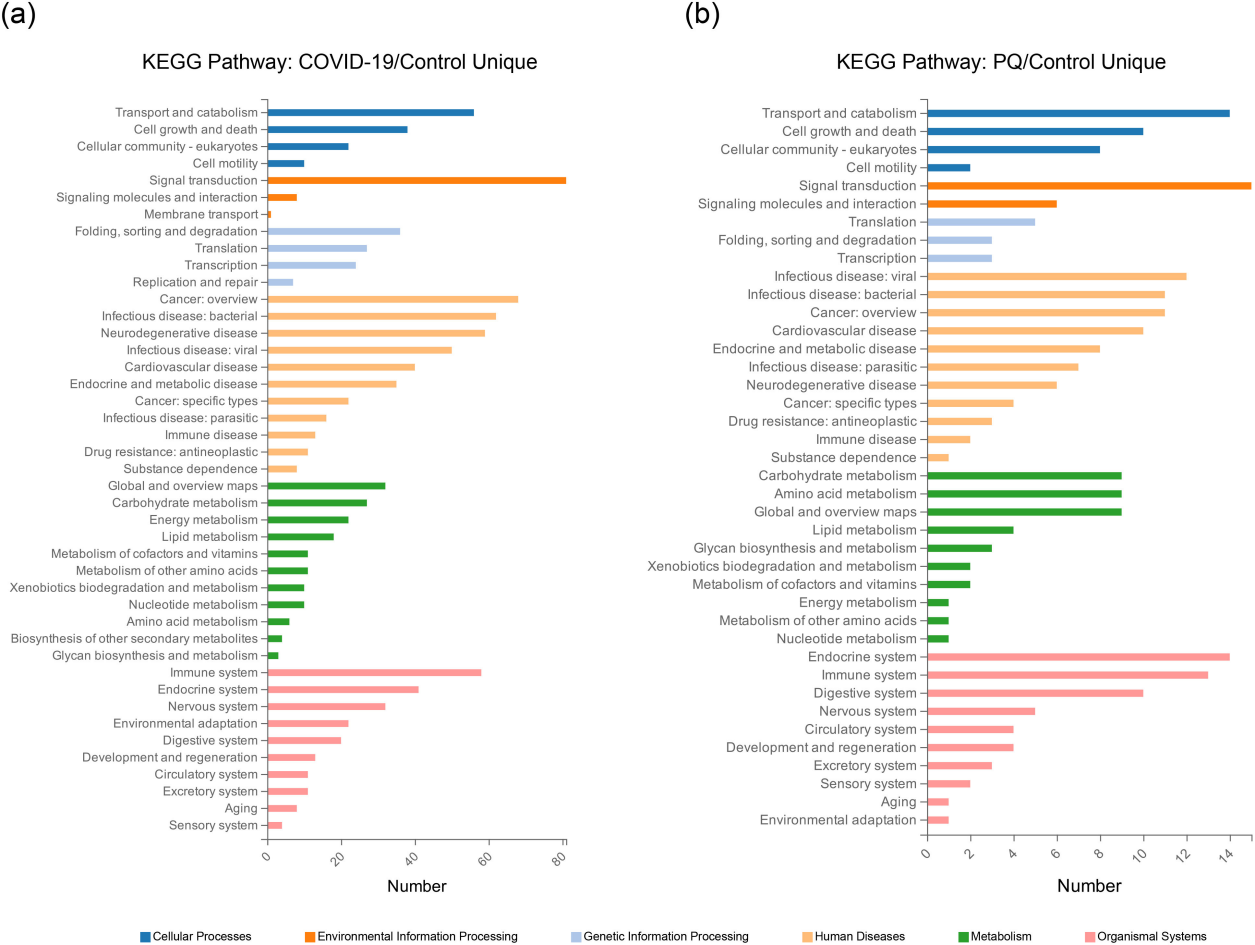
Unique DEPs for each of the two comparison groups for KEGG enrichment analysis. **(A)** KEGG Pathway enrichment analysis of the 816 DEPs unique to the COVID-19/Control comparison group. **(B)** KEGG Pathway enrichment analysis of the 141 DEPs unique to the PQ/Control comparison group.

Analyzing the overlapping relationships of these DEPs, the number of DEPs coenriched in the COVID-19 and PQ comparison groups was 516, whereas those unique to the COVID-19 comparison group was 816. The PQ comparison group was smaller, with only 141 unique DEPs. We analyzed unique DEPs for KEGG enrichment in the two comparison groups (**Figures 2A, B**).

### 3.2 Pathways and DEPs associated with pulmonary fibrosis

We focused on pathways relevant to PF and conducted KEGG enrichment analysis on DEPs, particularly on the Wnt, TGF-β, and Hedgehog pathways. These pathways have been reported to be associated with PF, and pathological sections have been generated for preliminary validation.

#### 3.2.1 Wnt signaling pathway and ubiquitination

Four DEPs (RAC1, CAMK2G, DAAM1, and RAC2) were enriched in the noncanonical Wnt pathway and two DEPs (TLE3 and CACYBP) were enriched in the canonical pathway in the COVID-19 comparison group. The number of significant DEPs from the Wnt noncanonical pathway was higher than that from the Wnt/β-catenin classical (canonical) pathway. This phenomenon was also observed in the PQ comparison group. Among the four significantly different proteins in the PQ group, SERPINF1 and Smad4 were from the canonical pathway, whereas NFATC2, and PLCB1 were from the Wnt/Ca2+ pathway.

**Figure 3:**
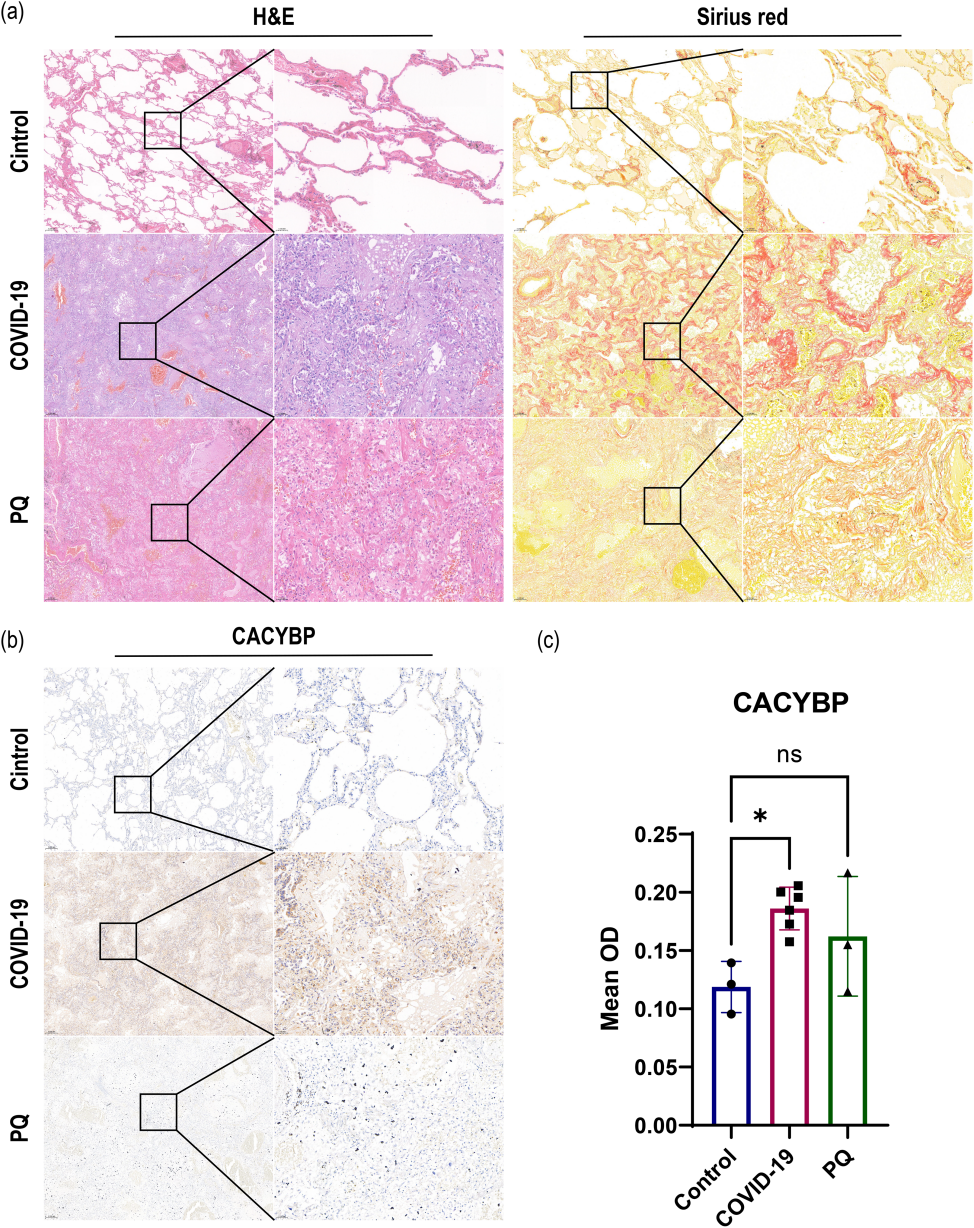
**(A)** H&E and Sirius red staining revealed severe pulmonary fibrosis in the COVID-19 and PQ groups. **(B, C)** IHC demonstrate that CACYBP is located in both the cytoplasm and the nucleus, with higher abundance observed in the COVID-19 comparison group than that in the PQ comparison group (Scale: 50× for low magnification, 200× for high magnification; *: P<0.05; ns: no significance).

In addition, we observed that the ubiquitination process in the Wnt/β-catenin classical (canonical) pathway warrants exploration owing to the alterations we observed in CACYBP in the proteomic data. The expression of CACYBP, involved in the regulation of nonphosphorylation-dependent ubiquitination, was upregulated in the COVID-19 comparison group. However, the difference was not significant in the PQ comparison group. To compare the differences in CACYBP expression between the two lung fibrosis groups, we conducted immunohistochemistry, H&E, and Sirius red staining of FFPE lung tissue samples from the three groups. IHC results for CACYBP showed its presence in the cytoplasm and nucleus. CACYBP abundance was higher in the COVID-19 comparison group than that in the PQ comparison group (**Figure 3**).

#### 3.2.2 TGF-β signaling pathway

The TGF-β signaling pathway plays a crucial role in the development of PF. The most significant DEP observed in the TGF-β pathway was the upregulation of FMOD in the COVID-19 and PQ comparison groups, with a more pronounced difference observed in the PQ group. Additionally, Smad4 exhibited significant downregulation in the PQ group, yet this difference was not significant in the COVID-19 comparison group. Another indicator enriched in TGF-β pathway differences, mitogen-activated protein kinase 1 (MAPK1), showed significant upregulation in the COVID-19 group. Nonetheless, the difference was not significant in the PQ group. Furthermore, TGF-β1, a central molecule in the TGF-β pathway, exhibited a Q-value > 0.05 in both groups compared with the control group despite having a Log2FC > 1. This suggests that TGF-β1 was upregulated in both groups of PF. However, the difference was not statistically significant.

#### 3.2.3 PI3K-Akt signaling pathway

More DEPs were enriched in the PI3K-Akt signaling pathway. Furthermore, 26 and 11 DEPs were found in the COVID-19 and PQ comparison groups. Initially, our focus was on identifying DEPs common to both comparison groups to determine the shared characteristics of lung fibrosis in both comparison groups. The common DEPs included TNC, FN1, MAP2K2, RELA, and EPHA2, which were upregulated, and ITGA2B downregulated in both comparison groups. TNC, FN1, and ITGA2B Q-values were < 0.005 for both comparison groups. We subsequently identified two groups of specific DEPs: PPP2R5E, SGK3, CDC37, TSC2, and 20 other DEPs that were upregulated- or downregulated solely in the COVID-19 group, with no observed differences in the PQ group. IKBKB, ITGA5, AKT1, COL6A3, and ITGA4 were upregulated solely in the PQ comparison group, with no difference observed in the COVID-19 comparison group (**Table 1**).

**Table 1:** One DEP in each group was enriched in pathways associated with pulmonary fibrosis.

Signaling Pathway	Symbol	Log2FC (COVID19/Control)	Qvalue (COVID19/Control)	Log2FC (PQ/Control)	Qvalue (PQ/Control)
Wnt	TLE3	2.334363041	0.001068722		
	PPP3CA	0.773500039	0.003699282	0.6622182	0.01814957
	RAC1	1.342190105	0.0129168		
	CAMK2G	1.152841075	0.01739589		
	DAAM1	-2.255988917	0.03492649		
	PLCB4	-0.970277022	0.03501096		
	RAC2	1.71137878	0.03606512		
	CACYBP	1.028170908	0.03758443		
	CTBP1	0.674958809	0.04614673		
	NFATC2	0.510556441	0.1327327	1.582644	0.0143101
	SERPINF1	0.817230288	0.05481924	1.052233	0.03618222
	PLCB1			-1.20737	0.03868567
	SMAD4			-1.040178	0.04678961
TGF-β	FMOD	1.525054828	0.01041436	2.231091	0.002736285
	MAPK1	0.570629695	0.0190492		
	SMAD4	0.07558513	0.8568159	-1.040178	0.04678961
PI3K-Akt	TNC	3.598133523	0.00000236	4.72209	0.000000801
	FN1	2.289614174	0.00005523	3.027061	0.00001898
	ITGA2B	-1.628314963	0.000300505	-1.235885	0.004655138
	PPP2R5E	2.469928867	0.000501024		
	BAD	2.710905434	0.000802183		
	SGK3	1.803091666	0.000895442		
	CDC37	1.323246163	0.001123117		
	TSC2	1.541233867	0.001862487		
	MAP2K2	3.062623437	0.00289972	2.921303	0.01187354
	HSP90AB1	1.791747126	0.00385903		
	GRB2	1.304040067	0.007280749		
	YWHAH	0.887984554	0.007413334		
	RAC1	1.342190105	0.0129168		
	GNB2	0.709724132	0.01713441		
	PKN2	-1.821455022	0.0187558		
	MAPK1	0.570629695	0.0190492		
	YWHAB	0.777029311	0.01932895		
	SYK	-0.913971923	0.02196084		
	EPHA2	1.352442423	0.02538842	1.211464	0.04666163
	CHUK	-1.789637666	0.02650397		
	NFKB1	1.140610106	0.02687618		
	MAP2K1	1.355991046	0.03054679		
	YWHAZ	0.606165866	0.03620794		
	ITGB3	-1.680240038	0.04077571		
	RELA	1.559710702	0.0416755	1.836263	0.03861816
	EIF4B	1.79392172	0.04528951		
	AKT1			1.113971	0.02168375
	ITGA5			1.510934	0.01126765
	IKBKB			2.243506	0.007500147
	ITGA4			1.839896	0.04634894
	COL6A3			1.932291	0.02196405
Hedgehog	ARRB1	1.090237398	0.04105257	1.295323	0.03666427
Notch1	TLE3	2.334363041	0.001068722		
	NCSTN	1.696530228	0.001345855	1.036378	0.03873798
	CTBP1	0.674958809	0.04614673		
	DTX3L			-1.377831	0.04540409
Hippo	PPP1CA	0.86048615	0.00279096		
	YWHAH	0.887984554	0.007413334		
	YWHAB	0.777029311	0.01932895		
	TP53BP2	-1.486384353	0.02616206		
	YWHAZ	0.606165866	0.03620794		
	SMAD4			-1.040178	0.04678961

A blank box illustrates undetected proteins that showed no statistically significant difference.

#### 3.2.4 Hedgehog, Hippo, and Notch pathways

ARRB1 exhibited significant upregulation in the COVID-19 and PQ comparison groups. Moreover, it was the only protein that was significantly enriched in the Hedgehog pathway. In addition, slightly more DEPs were enriched in the Hippo pathway. In the COVID-19 comparison group, five DEPs, PPP1CA, YWHAH, YWHAB, YWHAZ, and TP53BP2, were enriched with the first three upregulated and TP53BP2 downregulated, whereas the PQ group exhibited enrichment only in Smad4 within this pathway. Both genes were enriched in the Notch pathway: TLE3, NCSTN, and ARRB1. TP53BP2 and TP53BP2 were coenriched in the Notch pathway. Both genes were also coenriched in the Notch pathway, TLE3, and NCSTN; nonetheless, CTBP1 significantly differed in the COVID-19 comparison group, whereas DTX3L was more significant in the PQ comparison group.

**Table 1** illustrates the summary of manually screened pulmonary fibrosis-associated DEPs.

### 3.3 PPI of COVID-19 or PQ comparison group

**Figure 4:**
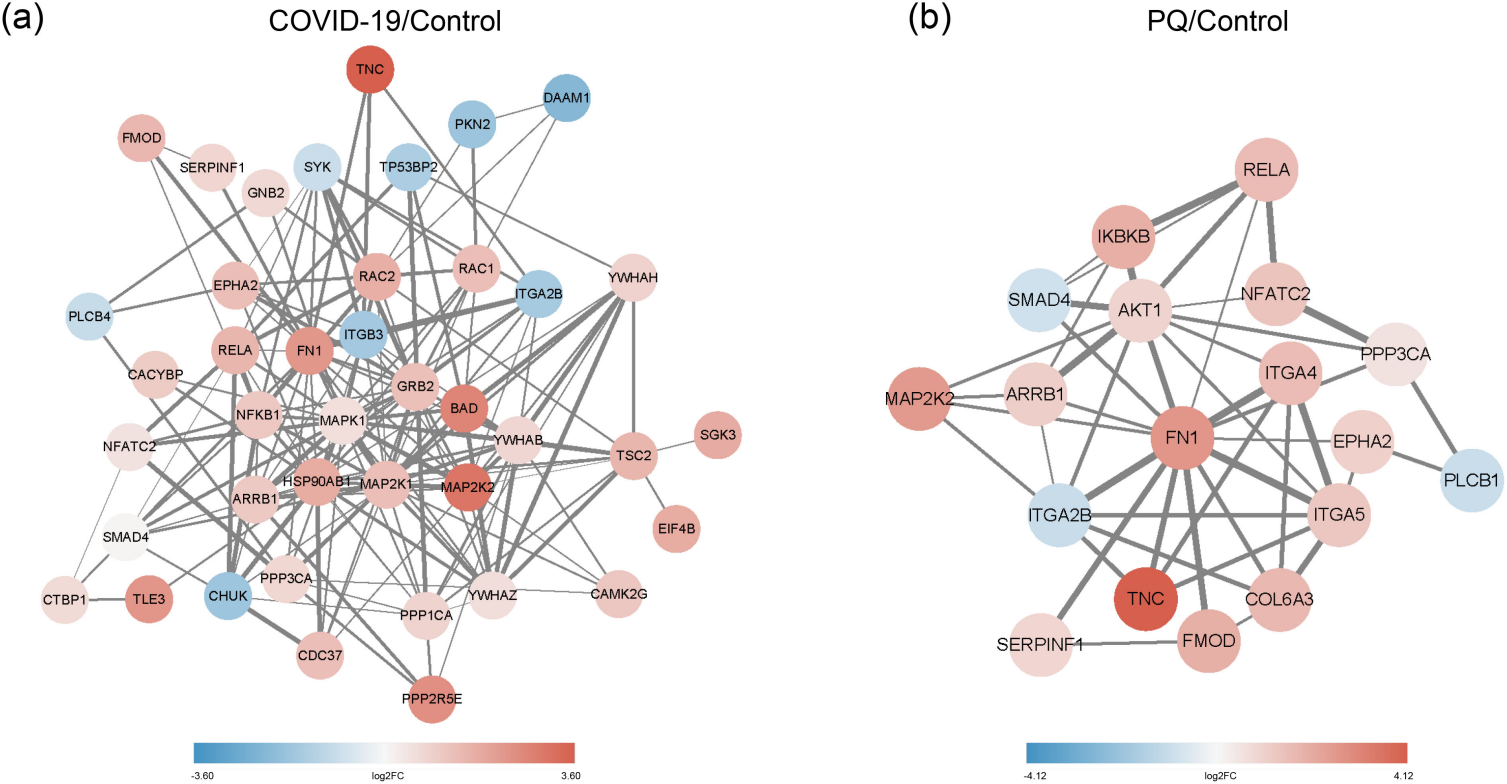
**(A)** PPI network of COVID-19/Control. **(B)** PPI network of PQ/Control. Network nodes depict proteins, while edges indicate protein-protein associations. Pink denotes upscaling, blue indicates downscaling, and the color intensity reflects the absolute value of log2FC. Edge thickness represents the degree of association between the paired nodes.

As identified above, the DEPs of lung fibrosis-related pathways were imported into String for PPI analysis. The minimum required interaction score was set to medium confidence (0.0400), and the meaning of network edges was defined as “confidence.” Simultaneously, we color-coded each node: pink for upscaling and blue for downscaling; the darker the color, the larger the absolute value of log2FC. In the COVID-19 group, the DEPS with the highest number of edges were MAPK1, MAP2K1, HSP90AB1, and FN1. The edge numbers are 23, 21, 20, and 19. Among them, FN1 exhibited the highest log2FC value. Moreover, SGK3 showed the smallest log2FC value among the downregulated proteins, with a node degree of one (**Figure 4A**).

In the PQ group, FN1 exhibited the highest numbers of edges with other DEPs, with a node degree of 14, followed by AKT1 with a node degree of 11. FN1 also displayed a significant variation. The figure shows the dark pink nodes (**Figure 4B**). The combination of TNC and FN1 appeared to play a significant role in both comparison groups.

## 4 Discussion

COVID-19 and PQ-induced PF exhibit similar types of lung fibrosis-related pathways. However, differences exist in the DEPs enriched within these pathways, as evidenced by human lung tissue sample data. BCL2-associated athanogene 2 (BAG2) functions as a potent and specific inhibitor of the ubiquitin ligase activity dependent on the carboxyl terminus of Hsp70-interacting protein (CHIP) (Dai et al., 2005). It plays a role in ER-associated degradation and inhibits the ubiquitin ligase activity of CHIP by disrupting the cooperation between CHIP/E2 (Arndt et al., 2005). The NEF function of BAG2 accelerates the ATPase cycle and can influence various cellular processes such as folding, aggregation, and degradation. In addition, BAG2 serves as an inhibitor of the Hsp70-binding E3 ubiquitin ligase microarray (carboxyl terminus of Hsp70-interacting proteins) (Qin et al., 2016). This suggests that the ubiquitination and subsequent degradation of at least one protein was hindered in lung tissue fibrosis following PQ exposure, while this effect was not observed in the COVID-19 group. Meanwhile, recent literature demonstrates that CTNNBL1 inhibits retroviral HIV-1 replication by suppressing the integration of viral DNA into the cellular genome.

CTNNBL1 is a nuclear protein expressed extensively, which binds to the Prp19 complex of the spliceosome and interacts with its CDC5L component (Conticello et al., 2008). Furthermore, recent literature highlights that CTNNBL1 can hinder the replication of retroviral HIV-1 by suppressing the integration of viral DNA into the cellular genome (Liang et al., 2022). In contrast, CTNNBL1 shares structural homology with CTNNB1 (also known as β-catenin) in the classical Wnt signaling pathway, suggesting a role either upstream or parallel to β-catenin (Huhn et al., 2011). Therefore, we hypothesize that CTNNBL1 is closely linked to retroviral replication, cell proliferation, and apoptosis.

Classical and nonclassical Wnt pathways are activated during pulmonary fibrogenesis (Chilosi et al., 2003). Identifying and validating the Wnt/Ca2+ pathway originates from investigations into the Wnt signaling that did not rely on β-catenin. Studies conducted on zebrafish and African clawed toads showed that Frizzled-2 triggered a more significant release of intracellular Ca2+ than Frizzled-1 (Kuhl et al., 2000). The induction of ECM expression through TGF-β, triggered by Ca2+, plays a crucial role in the progression of PF (Li et al., 2015). Four DEPs (RAC1, CAMK2G, DAAM1, and RAC2) were enriched in the noncanonical Wnt pathway and two DEPs (TLE3 and CACYBP) were enriched in the canonical pathway in the COVID-19 comparison group. A similar situation was observed in the PQ comparison group: two DEPs (PLCB1, and NFATC2) were enriched in the noncanonical Wnt pathway and two DEPs (SERPINF1 and SMAD4) were enriched in the canonical pathway, suggesting that the activation of the noncanonical Wnt pathway is not less effective than that of the canonical pathway in both types of lung fibrosis. This suggests that noncanonical pathways, which have often been overlooked in previous studies of lung fibrosis models, hold significant importance.

Ubiquitination-mediated degradation of β-catenin occurs through two pathways: phosphorylation- and nonphosphorylation-dependent. In the phosphorylation-dependent pathway, β-catenin is targeted for degradation by the destruction complex. This complex comprises Ser/Thr kinases such as glycogen synthase kinase 3 (GSK-3) and casein kinase 1 (CK1), along with the scaffolding protein Axin, the adenomatous polyposis coli (APC) protein, and the E3-ubiquitin ligase β-TrCP. First, β-catenin undergoes phosphorylation, which is degraded by the ubiquitin-proteasome system, facilitated by the Skp1/Cullin/Rbx1/F-box (SCF) E3 ubiquitin ligase complex (Orford et al., 1997; Kimbrel and Kung, 2009; Stamos and Weis, 2013). Ubiquitination, independent of phosphorylation, is linked to the p53 signaling pathway. Within this pathway, p53 triggers the expression of mammalian homologs of Drosophila Sina (Siah). Subsequently, Siah interacts with CACYBP (also known as SIP) and binds to Skp1, collectively facilitating the degradation of β-catenin. CACYBP serves as a molecular bridge, facilitating the formation of the ubiquitin E3 ligase and enabling interaction between Siah-1 and Skp1 (Matsuzawa and Reed, 2001; Fukushima et al., 2006). Negative feedback is an excellent protective mechanism in response to foreign microbial invasion in organisms. The phosphorylation-dependent pathway of β-catenin is feedback-activated during SARS-COV-2-induced damage to the lungs and leads to pulmonary fibrosis. This pathway is used for lung tissue protection and repair. Notably, CACYBP is specifically upregulated in SARS-COV-2 and not other viruses, take influenza virus and SARS for example. Xiao-Sheng Jiang et al. performed proteomics of SARS-COV as early as 2005 and detected only 52 DEPs (fold change>=2.0). There was no CACYBP among them that we detected (Jiang et al., 2005), but it is possible that proteomics technology was not as sophisticated as it is now. In 2021, Jinming Zhang et al. proposed that ubiquitination between influenza virus and host has ubiquitin-like modifiers such as SUMO, NEDD8, and ISG15, in addition to ubiquitin; whereas they did not report on our proposed CACYBP (Zhang et al., 2020). An article published in Nature Communications in 2022 elucidated the PPI network of influenza viruses interacting with host cells, but also did not report on CACYBP (Haas et al., 2023). Therefore, we hypothesize that CACYBP is a protein that is specifically up-regulated after SARS-COV-2 infection. Notably, several of the above articles used human-derived cell line samples for proteomic analyses, rather than autopsy samples, which may explain the discrepancy. Subsequently, if the conditions permit, we can perform CACYBP knockout in A549 cell lines or in mice and infect them with SARS-COV-2 or other viruses, and use cell crawls or pathological sections to observe the pathological changes, potentially linking the protein changes with pathological phenomena.

The TGF-β/Smad signaling pathway has been demonstrated to promote the synthesis of fibrinogen activator-1 (PAI-1) in SARS-CoV (Zhao et al., 2008). Moreover, TGF-β induces epithelial-to-mesenchymal transitions (EMTs), contributing to PF (Su et al., 2020). In the TGF-β/Smad signaling pathway, Smad4 binds to activated Smad2/Smad3 and regulates the transcription of target genes (Xia et al., 2015). Knockdown of Smad4 in thylakoid cells has been shown to inhibit TGF-β1-induced ECM deposition (Tsuchida et al., 2003). MAPK1 exhibits significant upregulation and enrichment for the TGF-β/Smad signaling pathway in the COVID-19 comparison group. This could be attributed to the cross-regulation of various pathways that collectively influence the depositional effects of ECM. This finding may explain the significant downregulation of Smad4 in the PQ comparison group.

The Phosphoinositide 3-kinase (PI3K)-AKT signaling network is activated under physiological conditions in response to growth and transcription factors. It regulates cellular metabolic processes and systemic metabolic homeostasis. Oncogenic activation of the PI3K-AKT pathway in cancer cells leads to a reprogramming of cellular metabolism. This involves enhancing the activity of nutrient transporters and metabolic enzymes to meet the anabolic demands of rapidly proliferating cells (Hoxhaj and Manning, 2020). Xiaoting Hu et al. initially highlighted *in vitro* and *in vivo* studies that the PI3K-Akt-mTOR/PFKFB3 pathway mediates collagen synthesis in PF. This occurs through the direct induction of aerobic glycolysis in lung fibroblasts by lipopolysaccharides (Hu et al., 2020). This study revealed an increased abundance of DEPs in the PI3K-AKT pathway. This suggests their significant involvement role in both types of lung fibrosis but with distinct foci.

The core protein of the Hippo pathway, YAP, facilitates the proliferation, migration, and collagen deposition of lung fibroblasts in response to mechanical signals in the respiratory system (Tang et al., 2022). This transition leads fibroblasts from a relatively quiescent state to a pathologically activated one, thereby promoting lung fibrosis (Dey et al., 2020). This highlights the complex crosstalk between Hippo and other signaling pathways. This is further supported by the enrichment of Smad4 from the TGF-β/Smads pathway within the Hippo pathway in the PQ comparison group. YWHAH, YWHAB, and YWHAZ, all members of 14-3-3ζ, can bind to YAP, thereby facilitating tissue regeneration and repair (Wang et al., 2023). TP53BP2 (ASPP2) plays a crucial role in p53-dependent and independent pathways as well as interact with phosphatase 1 (PP1) to regulate the phosphorylation of YAP (Huo et al., 2023).

β-arrestin1 (ARRB1) belongs to the β-arrestins family, which is recognized for its role in regulating G protein (heterotrimeric guanine nucleotide-binding protein)–coupled receptor (GPCR) signaling through receptor desensitization and internalization (DeWire et al., 2007). ARRB1 has consistently been associated with hepatic fibrosis. Its transcripts and proteins were upregulated during murine toxin-induced hepatic fibrosis (Yang et al., 2015). In a constitutive ARRB1 knockout mouse model of hepatic fibrosis, collagen deposition in the liver was decreased by 50% (Yang et al., 2015). In contrast, Alysia et al. discovered that ARRB1 deletion led to a reduction in bleomycin-induced PF. In this model, ARRB1 deletion did not affect the responsiveness of fibroblasts to TGF-β (Lovgren et al., 2011). This suggests that the function of ARRB1 is not dependent on the established TGF-β pathway. Alternatively, its increased expression across various types of PF suggests that ARRB1 may contribute to a shared biological mechanism during the progression of PF induced by diverse factors (both pathological and toxic). Thus, it plays a crucial role in regulating collagen and ECM formation.

In the PPI analysis data derived from the screened DEPs, FN1 (Fibronectin1) stood out as the DEP significantly upregulated in both groups, exhibiting a high node degree. FN is a multifunctional extracellular matrix protein and a high-molecular-weight glycoprotein. It binds to various components on the cell surface and in the extracellular matrix, including collagen, fibronectin, heparin, DNA and actin, among others (Patten and Wang, 2021). FN1 serves as a biomarker of EMT and is often upregulated in many tumors (Liu et al., 2020b).

In this study, only three cases of pulmonary fibrosis were observed to have been caused by PQ poisoning, which benefited from the restrictions imposed by the Chinese government on the use of PQ. However, it also limits the number of samples. Furthermore, our samples were collected from patients infected with the original SARS-COV-2 strain, and we did not retain frozen tissue samples due to biosecurity issues, which prevented us from adding Western-blot (WB) images. In future studies, if circumstances allow, we suggest that researchers can explore more advanced detection methods to gather additional data and insights, such as intrinsic fluorescence imaging (IFI). In addition, this study lacks the support of results from animal or cell models, we aim to continue our study in the direction later.

In conclusion, this study highlights the potential therapeutic targets, such as CACYBP, which may offer new avenues for developing targeted interventions for PF. Relevant pathways associated with PF reported in the literature were examined. This approach sheds light on the similarities and differences between pathological and toxic PF, offering insights for further exploration. In future studies, we aim to focus on elucidating the specific functions of proteins such as CACYBP in the context of PF induced by different factors. Understanding the molecular mechanisms underlying their involvement could provide insights into potential therapeutic targets. Moreover, the role of the non-canonical Wnt pathway in the two pulmonary fibrosis processes is as important as the classical pathway; hence, such non-canonical pathways also need to be studied. The exploration of signaling pathways also plays an important role. The crosstalk between signaling pathways implicated in PF, such as the Wnt/Ca2+, TGF-β/Smad, and PI3K-AKT pathways. Understanding how these pathways interact and influence each other could uncover novel therapeutic strategies for treating pulmonary fibrosis.

## Data Availability

The datasets presented in this study can be found in online repositories. The names of the repository/repositories and accession number(s) can be found in the article/**Supplementary Material**.

## References

[B1] Arndt V. Daniel C. Nastainczyk W. Alberti S. Höhfeld J. (2005). BAG-2 acts as an inhibitor of the chaperone-associated ubiquitin ligase CHIP. Mol. Biol. Cell 16, 5891–5900. doi: 10.1091/mbc.e05-07-0660 16207813 PMC1289430

[B2] Borczuk A. C. Salvatore S. P. Seshan S. V. Patel S. S. Bussel J. B. Mostyka M.. (2020). COVID-19 pulmonary pathology: a multi-institutional autopsy cohort from Italy and New York City. Mod Pathol. 33, 2156–2168. doi: 10.1038/s41379-020-00661-1 32879413 PMC7463226

[B3] Chilosi M. Poletti V. Zamò A. Lestani M. Montagna L. Piccoli P.. (2003). Aberrant wnt/β-catenin pathway activation in idiopathic pulmonary fibrosis. Am. J. Pathol. 162, 1495–1502. doi: 10.1016/S0002-9440(10)64282-4 12707032 PMC1851206

[B4] Choi M. Chang C.-Y. Clough T. Broudy D. Killeen T. Maclean B.. (2014). MSstats: an R package for statistical analysis of quantitative mass spectrometry-based proteomic experiments. Bioinformatics 30, 2524–2526. doi: 10.1093/bioinformatics/btu305 24794931

[B5] Conticello S. G. Ganesh K. Xue K. Lu M. Rada C. Neuberger M. S. (2008). Interaction between antibody-diversification enzyme AID and spliceosome-associated factor CTNNBL1. Mol. Cell 31, 474–484. doi: 10.1016/j.molcel.2008.07.009 18722174

[B6] Dai Q. Qian S.-B. Li H.-H. McDonough H. Borchers C. Huang D.. (2005). Regulation of the cytoplasmic quality control protein degradation pathway by BAG2. J. Biol. Chem. 280, 38673–38681. doi: 10.1074/jbc.M507986200 16169850

[B7] DeWire S. M. Ahn S. Lefkowitz R. J. Shenoy S. K. (2007). Beta-arrestins and cell signaling. Annu. Rev. Physiol. 69, 483–510. doi: 10.1146/annurev.physiol.69.022405.154749 17305471

[B8] Dey A. Varelas X. Guan K. L. (2020). Targeting the Hippo pathway in cancer, fibrosis, wound healing and regenerative medicine. Nat. Rev. Drug Discovery 19, 480–494. doi: 10.1038/s41573-020-0070-z 32555376 PMC7880238

[B9] Dinis-Oliveira R. J. Duarte J. A. Sanchez-Navarro A. Remiao F. Bastos M. L. Carvalho F. (2008). Paraquat poisonings: mechanisms of lung toxicity, clinical features, and treatment. Crit. Rev. Toxicol. 38, 13–71. doi: 10.1080/10408440701669959 18161502

[B10] Fukushima T. Zapata J. M. Singha N. C. Thomas M. Kress C. L. Krajewska M.. (2006). Critical function for SIP, a ubiquitin E3 ligase component of the beta-catenin degradation pathway, for thymocyte development and G1 checkpoint. Immunity 24, 29–39. doi: 10.1016/j.immuni.2005.12.002 16413921

[B11] Gao F. Zhang Y. Yang Z. Wang M. Zhou Z. Zhang W.. (2020). Arctigenin suppressed epithelial-mesenchymal transition through Wnt3a/beta-catenin pathway in PQ-induced pulmonary fibrosis. Front. Pharmacol. 11, 584098. doi: 10.3389/fphar.2020.584098 33390951 PMC7772408

[B12] Gawarammana I. B. Buckley N. A. (2011). Medical management of paraquat ingestion. Br. J. Clin. Pharmacol. 72, 745–757. doi: 10.1111/j.1365-2125.2011.04026.x 21615775 PMC3243009

[B13] George P. M. Wells A. U. Jenkins R. G. (2020). Pulmonary fibrosis and COVID-19: the potential role for antifibrotic therapy. Lancet Respir. Med. 8, 807–815. doi: 10.1016/S2213-2600(20)30225-3 32422178 PMC7228727

[B14] Haas K. M. McGregor M. J. Bouhaddou M. Polacco B. J. Kim E.-Y. Nguyen T. T.. (2023). Proteomic and genetic analyses of influenza A viruses identify pan-viral host targets. Nat. Commun. 14, 6030. doi: 10.1038/s41467-023-41442-z 37758692 PMC10533562

[B15] Henderson W. R. Jr. Chi E. Y. Ye X. Nguyen C. Tien Y. T. Zhou B.. (2010). Inhibition of Wnt/beta-catenin/CREB binding protein (CBP) signaling reverses pulmonary fibrosis. Proc. Natl. Acad. Sci. U.S.A. 107, 14309–14314. doi: 10.1073/pnas.1001520107 20660310 PMC2922550

[B16] Hinz B. Phan S. H. Thannickal V. J. Galli A. Bochaton-Piallat M. L. Gabbiani G. (2007). The myofibroblast: one function, multiple origins. Am. J. Pathol. 170, 1807–1816. doi: 10.2353/ajpath.2007.070112 17525249 PMC1899462

[B17] Hoxhaj G. Manning B. D. (2020). The PI3K-AKT network at the interface of oncogenic signaling and cancer metabolism. Nat. Rev. Cancer 20, 74–88. doi: 10.1038/s41568-019-0216-7 31686003 PMC7314312

[B18] Hu X. Xu Q. Wan H. Hu Y. Xing S. Yang H.. (2020). PI3K-Akt-mTOR/PFKFB3 pathway mediated lung fibroblast aerobic glycolysis and collagen synthesis in lipopolysaccharide-induced pulmonary fibrosis. Lab. Invest. 100, 801–811. doi: 10.1038/s41374-020-0404-9 32051533

[B19] Huang W. J. Tang X. X. (2021). Virus infection induced pulmonary fibrosis. J. Transl. Med. 19, 496. doi: 10.1186/s12967-021-03159-9 34876129 PMC8649310

[B20] Huhn S. Ingelfinger D. Bermejo J. L. Bevier M. Pardini B. Naccarati A.. (2011). Polymorphisms in CTNNBL1 in relation to colorectal cancer with evolutionary implications. Int. J. Mol. Epidemiol. Genet. 2, 36–50. 21537400 PMC3077237

[B21] Huo Y. Cao K. Kou B. Chai M. Dou S. Chen D.. (2023). TP53BP2: Roles in suppressing tumorigenesis and therapeutic opportunities. Genes Dis. 10, 1982–1993. doi: 10.1016/j.gendis.2022.08.014 37492707 PMC10363587

[B22] Jiang X.-S. Tang L.-Y. Dai J. Zhou H. Li S.-J. Xia Q.-C.. (2005). Quantitative analysis of severe acute respiratory syndrome (SARS)-associated coronavirus-infected cells using proteomic approaches. Mol. Cell. Proteomics 4, 902–913. doi: 10.1074/mcp.M400112-MCP200 15784933 PMC7780044

[B23] Jin H. (2020). Imrecoxib inhibits paraquat-induced pulmonary fibrosis through the NF-kappaB/snail signaling pathway. Comput. Math Methods Med. 2020, 6374014. doi: 10.1155/2020/6374014 33123215 PMC7582077

[B24] Kimbrel E. A. Kung A. L. (2009). The F-box protein beta-TrCp1/Fbw1a interacts with p300 to enhance beta-catenin transcriptional activity. J. Biol. Chem. 284, 13033–13044. doi: 10.1074/jbc.M901248200 19297328 PMC2676036

[B25] Konigshoff M. Balsara N. Pfaff E. M. Kramer M. Chrobak I. Seeger W.. (2008). Functional Wnt signaling is increased in idiopathic pulmonary fibrosis. PloS One 3, e2142. doi: 10.1371/journal.pone.0002142 18478089 PMC2374879

[B26] Kuhl M. Sheldahl L. C. Park M. Miller J. R. Moon R. T. (2000). The Wnt/Ca2+ pathway: a new vertebrate Wnt signaling pathway takes shape. Trends Genet. 16, 279–283. doi: 10.1016/s0168-9525(00)02028-x 10858654

[B27] Kumar S. Gupta S. Bansal Y. S. Bal A. Rastogi P. Muthu V.. (2021). Pulmonary histopathology in fatal paraquat poisoning. Autops Case Rep. 11, e2021342. doi: 10.4322/acr.2021.342 34926332 PMC8676609

[B28] Li C. Bellusci S. Borok Z. Minoo P. (2015). Non-canonical WNT signaling in the lung. J. Biochem. 158, 355–365. doi: 10.1093/jb/mvv081 26261051 PMC4751232

[B29] Liang G. He Y. Zhao L. Ouyang J. Geng W. Zhang X.. (2022). CTNNBL1 restricts HIV-1 replication by suppressing viral DNA integration into the cell genome. Cell Rep. 38, 110533. doi: 10.1016/j.celrep.2022.110533 35294870

[B30] Liu Q. Shi Y. Cai J. Duan Y. Wang R. Zhang H.. (2020a). Pathological changes in the lungs and lymphatic organs of 12 COVID-19 autopsy cases. Natl. Sci. Rev. 7, 1868–1878. doi: 10.1093/nsr/nwaa247 34676085 PMC7543449

[B31] Liu X. Meng L. Li X. Li D. Liu Q. Chen Y.. (2020b). Regulation of FN1 degradation by the p62/SQSTM1-dependent autophagy–lysosome pathway in HNSCC. Int. J. Oral. Sci. 12 (1), 34. doi: 10.1038/s41368-020-00101-5 33318468 PMC7736930

[B32] Lovgren A. K. Kovacs J. J. Xie T. Potts E. N. Li Y. Foster W. M.. (2011). beta-arrestin deficiency protects against pulmonary fibrosis in mice and prevents fibroblast invasion of extracellular matrix. Sci. Transl. Med. 3, 74ra23. doi: 10.1126/scitranslmed.3001564 PMC3094726 21411739

[B33] Matsuzawa S. I. Reed J. C. (2001). Siah-1, SIP, and Ebi collaborate in a novel pathway for beta-catenin degradation linked to p53 responses. Mol. Cell 7, 915–926. doi: 10.1016/S1097-2765(01)00242-8 11389839

[B34] Moss B. J. Ryter S. W. Rosas I. O. (2022). Pathogenic mechanisms underlying idiopathic pulmonary fibrosis. Annu. Rev. Pathol. 17, 515–546. doi: 10.1146/annurev-pathol-042320-030240 34813355

[B35] Nalbandian A. Sehgal K. Gupta A. Madhavan M. V. McGroder C. Stevens J. S.. (2021). Post-acute COVID-19 syndrome. Nat. Med. 27, 601–615. doi: 10.1038/s41591-021-01283-z 33753937 PMC8893149

[B36] Orford K. Crockett C. Jensen J. P. Weissman A. M. Byers S. W. (1997). Serine phosphorylation-regulated ubiquitination and degradation of beta-catenin. J. Biol. Chem. 272, 24735–24738. doi: 10.1074/jbc.272.40.24735 9312064

[B37] Patten J. Wang K. (2021). Fibronectin in development and wound healing. Advanced Drug Delivery Rev. 170, 353–368. doi: 10.1016/j.addr.2020.09.005 32961203

[B38] Qin L. Guo J. Zheng Q. Zhang H. (2016). BAG2 structure, function and involvement in disease. Cell. Mol. Biol. Lett. 21, 18. doi: 10.1186/s11658-016-0020-2 28536620 PMC5415834

[B39] Rosenbloom J. Macarak E. Piera-Velazquez S. Jimenez S. A. (2017). Human fibrotic diseases: current challenges in fibrosis research. Methods Mol. Biol. 1627, 1–23. doi: 10.1007/978-1-4939-7113-8_1 28836191

[B40] Stamos J. L. Weis W. I. (2013). The beta-catenin destruction complex. Cold Spring Harb. Perspect. Biol. 5, a007898. doi: 10.1101/cshperspect.a007898 23169527 PMC3579403

[B41] Su J. Morgani S. M. David C. J. Wang Q. Er E. E. Huang Y. H.. (2020). TGF-beta orchestrates fibrogenic and developmental EMTs via the RAS effector RREB1. Nature 577, 566–571. doi: 10.1038/s41586-019-1897-5 31915377 PMC7450666

[B42] Tang W. Li M. Yangzhong X. Zhang X. Zu A. Hou Y.. (2022). Hippo signaling pathway and respiratory diseases. Cell Death Discovery 8, 213. doi: 10.1038/s41420-022-01020-6 35443749 PMC9021242

[B43] Thannickal V. J. Toews G. B. White E. S. Lynch J. P. 3rd Martinez F. J. (2004). Mechanisms of pulmonary fibrosis. Annu. Rev. Med. 55, 395–417. doi: 10.1146/annurev.med.55.091902.103810 14746528

[B44] Tsuchida K. Zhu Y. Siva S. Dunn S. R. Sharma K. (2003). Role of Smad4 on TGF-beta-induced extracellular matrix stimulation in mesangial cells. Kidney Int. 63, 2000–2009. doi: 10.1046/j.1523-1755.2003.00009.x 12753287

[B45] Vannella K. M. Wynn T. A. (2017). Mechanisms of organ injury and repair by macrophages. Annu. Rev. Physiol. 79, 593–617. doi: 10.1146/annurev-physiol-022516-034356 27959618

[B46] Wang T. T. Wu L. L. Wu J. Zhang L. S. Shen W. J. Zhao Y. H.. (2023). 14-3-3zeta inhibits maladaptive repair in renal tubules by regulating YAP and reduces renal interstitial fibrosis. Acta Pharmacol. Sin. 44, 381–392. doi: 10.1038/s41401-022-00946-y 35840657 PMC9889378

[B47] WHO Coronavirus (COVID-19) Dashboard. Available online at: https://covid19.who.int/ (Accessed 31 March 2024).

[B48] Xia X. Wu W. Huang C. Cen G. Jiang T. Cao J.. (2015). SMAD4 and its role in pancreatic cancer. Tumor Biol. 36, 111–119. doi: 10.1007/s13277-014-2883-z 25464861

[B49] Yadav R. K. Gurung S. Karki S. Lama S. Tamang S. Poudel M. (2023). Acute paraquat poisoning complicated by acute kidney injury and lung fibrosis: a case report from Nepal. Ann. Med. Surg. (Lond) 85, 5117–5119. doi: 10.1097/MS9.0000000000001166 37811118 PMC10553043

[B50] Yang Y. Guo Y. Tan S. Ke B. Tao J. Liu H.. (2015). beta-Arrestin1 enhances hepatocellular carcinogenesis through inflammation-mediated Akt signaling. Nat. Commun. 6, 7369. doi: 10.1038/ncomms8369 26077142

[B51] Zhang J. Peng Q. Zhao W. Sun W. Yang J. Liu N. (2020). Proteomics in influenza research: the emerging role of posttranslational modifications. J. Proteome Res. 20, 110–121. doi: 10.1021/acs.jproteome.0c00778 33348980

[B52] Zhao X. Nicholls J. M. Chen Y. G. (2008). Severe acute respiratory syndrome-associated coronavirus nucleocapsid protein interacts with Smad3 and modulates transforming growth factor-beta signaling. J. Biol. Chem. 283, 3272–3280. doi: 10.1074/jbc.M708033200 18055455 PMC8740907

